# Current and Novel Treatment Modalities of Idiopathic Pulmonary Fibrosis

**DOI:** 10.7759/cureus.56140

**Published:** 2024-03-14

**Authors:** Mahnoor Arshad, Zoraize Moeez Athar, Tasneem Hiba

**Affiliations:** 1 Internal Medicine, BronxCare Health System, Bronx, USA

**Keywords:** pde 4b inhibitor, ipf, lung transplant, pirfenidone, nintedanib, treatment, idiopathic pulmonary fibrosis

## Abstract

Idiopathic pulmonary fibrosis (IPF) presents a clinical challenge characterized by progressive fibrosis and destruction of lung tissue. Despite recent advancements, including antifibrotic medications like pirfenidone and nintedanib, IPF remains a chronic and often fatal condition with limited treatment options. This article provides an overview of the current treatment modalities for IPF and explores the need for new therapeutic approaches. Antifibrotic medications have shown efficacy in slowing disease progression but are not curative and may not be suitable for all patients. Ongoing research focuses on emerging therapies such as stem cell therapy, immunomodulatory agents, and novel pharmacological targets like phosphodiesterase 4B (PDE4B) inhibitors. While these treatments offer promise, there remains an unmet need for effective therapies capable of halting or reversing fibrotic lung damage.

## Introduction and background

Idiopathic pulmonary fibrosis (IPF) is the most common interstitial lung disease (ILD) [1]. It is characterized by fibrosis, inflammation, and eventual destruction of the lung architecture. The main pathogenesis theorized is damage to the alveolar epithelium and abnormal wound repair. It is a progressive, chronic condition with a poor long-term prognosis. Chances of survival from 5 years of diagnosis range between 20-40% [2]. About 70% of this demographic is male with cigarette smoking identified as a common correlation [3]. Diagnosing IPF can present a clinical dilemma for clinicians, however, in an immunocompetent individual, the presence of all of the following major criteria and three of four minor criteria increases the chances of a correct diagnosis [4]. 

Major criteria: 1) Effectively ruling out other known causes of ILD; 2) Pulmonary function testing (PFT) demonstrating restrictive pattern; 3) High-resolution computed tomography (HRCT) scans significant for bibasilar reticular abnormalities with minimal ground glass opacities; 4) Transbronchial lung biopsy ruling out other alternative diagnoses.

Minor criteria: 1) Duration of illness >3 months; 2) age at onset >50 years; 3) unexplained causes of dyspnea at exertion; 4) bibasilar inspiratory crackles.

Pathogenesis 

The pathogenesis of IPF has been subject to much debate over the centuries. There is growing evidence that IPF may represent a separate disorder in which fibrogenesis results, at least in part, from multi-focal epithelial micro-injury. Repeated injury to the alveolar epithelial cell (AEC) leads to apoptosis, which might lead to disordered epithelial-fibroblastic interactions and aberrant repair processes, finally resulting in fibrosis [5]. It is generally acknowledged that repeated injury is caused by an interaction between genetic predisposition and injurious environmental agents. 

Genetic Factors

Interestingly, familial clustering of IPF cases has been reported implicating a genetic predisposition. New research includes the characterization of the *ELMOD2 *gene and the role of surfactant protein (SP)-C. *ELMOD2 *mRNA expression is significantly decreased in IPF lungs in comparison with healthy control subjects. *ELMOD2 *has been shown to be implicated in the regulation of interferon-related antiviral responses [6]. 

Injurious Agents

Multiple hit hypotheses have been proposed to understand the underlying pathogenesis of IPF, which signifies the association of a genetic predisposition to abnormal epithelial cell regulation with environmental triggers. So far, the best-known environmental factors recognized are viruses and gastroesophageal reflux. 

Viruses implicated in the pathogenesis of IPF include Epstein-Barr virus (EBV), human herpes viruses 7 and 8, cytomegalovirus, hepatitis C virus, herpes simplex virus, parvovirus B19, and torque teno virus [7]. EBV infection is frequently found in IPF patients, both in familial and sporadic cases. EBV has been detected in the lung tissue of IPF patients, particularly in the AECs [8,9]. The expression of certain EBV proteins in AEC is associated with a poorer prognosis in IPF [10]. Treatment with oral antiviral therapy has shown some clinical stability in IPF patients. EBV-infected cells exposed to transforming growth factor Beta-1 (TGF-β1), a key cytokine in IPF, display viral activation and resistance to growth inhibition [11]. Therapies targeting TGF-β1 are now under study as potential treatments for IPF. 

A study by Tcherakian et al. has provided evidence for the role of gastroesophageal reflux in the pathogenesis of both IPF and acute exacerbations. The researchers found that gastroesophageal reflux was more prevalent in patients with asymmetrical IPF compared to those with symmetrical IPF. Additionally, patients with asymmetrical IPF experienced a higher frequency of acute exacerbations than those with symmetrical IPF. The authors suggest that the observed asymmetry may indicate the involvement of factors such as gastroesophageal reflux or specific regional conditions in the development and progression of pulmonary fibrosis and acute exacerbations [12]. 

Prospective risk factors 

Cigarette smoking has been identified as a potential risk factor from multiple case-controlled studies with an odds ratio of 1.6 to 2.9 in every smoker around the world [13,14]. A study in the US revealed that those with a history of smoking for 21 to 40 pack-years had an OR of 2.3 (95% confidence interval (CI), 1.3 to 3.8) [4]. 

Commonly Prescribed Drugs

One case-control study has found an association between IPF and anti-depressants [4], however, the significance is unknown. 

Chronic Aspiration

Gastroesophageal reflux has been implicated in the development of IPF, however, the role in the pathogenesis of IPF is unclear. 

Infectious Agents

As discussed agents, numerous viruses, including EBV, have been studied to drive the pathogenesis of IPF. Additionally, mycoplasma and legionnaires disease have also been implicated [4]. 

Genetic Risk Factors

Genetic predisposition to IPF has been noted through studies on families with several affected members. In a Mexican case-control study, the presence of a family history of pulmonary fibrosis was found to be the most important risk factor for IPF with an odds ratio of 6.1 (95% confidence interval, 2.3-15.9) on multivariate analysis [15]. 

Despite the identification of various risk factors, the progressive nature and extensive remodeling involved in IPF remains unexplained and an understudied area in modern medicine. 

Clinical presentation

Dyspnea has been reported to be the most prevalent symptom present at the initial visit in a patient with IPF. There have been several studies which correlate between the severity of dyspnea and quality of life and survival in patients with IPF [16]. Another common manifestation of the disease is cough which is more common in nonsmokers and in advanced disease. Additionally, cough is also considered a predictor of disease progression [17]. On physical examination, fine crackles, predominantly in the lower posterior lung zones, are commonly reported in patients with IPF, and clubbed fingers are reported in 30-50% of patients. 

Routine spirometry reveals a restrictive pattern with decreased lung volumes and a decrease in diffusing capacity. As the severity of the disease progresses, forced expiratory volume in one second/FVC (forced vital capacity) increases, and diffusing capacity of the lungs for carbon monoxide (DLCO) decreases. An important predictor of mortality is a decline in FVC over a period of 6 to 12 months and a decline in DLCO has also been associated with decreased survival [18-20]. 

HRCT Findings

A usual interstitial pneumonia (UIP) pattern on HRCT, in an appropriate clinical setting, is diagnostic of IPF [18,21] HRCT findings of UIP consistent with IPF include subpleural, basal reticular abnormalities in the presence of honeycombing, with or without traction bronchiectasis, and the absence of any of inconsistent features [18]. Inconsistent features include upper or mid-lung predominance, peribronchovascular predominance, extent of ground-glass abnormality greater than reticular abnormality, profuse micronodules (bilateral, predominantly upper lobes), discrete cysts (multiple, bilateral, away from areas of honeycombing), diffuse mosaic attenuation/air-trapping (bilateral, in three or more lobes), or consolidation in bronchopulmonary segment(s)/lobe(s). 

**Figure 1:**
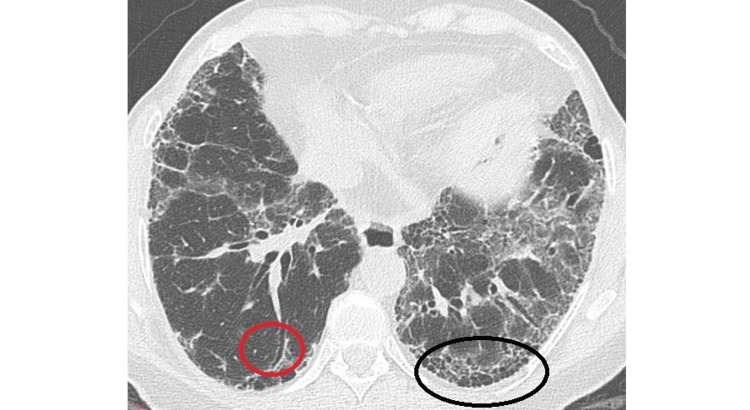
Definite usual interstitial pneumonia (UIP) pattern showing sub-pleural basal honeycombing (black circle) with traction bronchiectasis (red circle) Source: Sverzellati [22]

**Figure 2:**
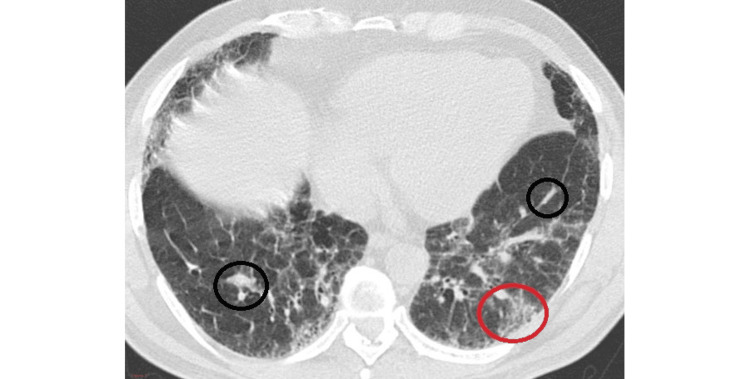
High-resolution computed tomography pattern inconsistent with UIP – demonstrating patchy ground glass (red circle) and peripheral reticular opacities (black circles) Source: Sverzellati [22]

The following figures demonstrate a definite UIP pattern (Figure 1) and an HRCT pattern inconsistent with UIP (Figure 2) [22].

While UIP patterns are commonly associated with IPF, there are instances where HRCT findings may not align with histopathological evidence. In such scenarios, it becomes crucial to explore alternative diagnoses such as systemic sclerosis-related ILD, rheumatoid arthritis-related ILD, connective tissue disease-ILD, and pulmonary sarcoidosis [23]. These alternative diagnoses present challenges and may indicate worse prognoses for ILD [23]. However, when both HRCT and histologic findings are consistent with UIP, the diagnosis of IPF is confirmed [23].

## Review

Current treatment modalities are diversified from pharmacotherapy to lung transplantation, as summarized in Table 1 [1,3,4].

**Table 1:** Current treatment modalities

Treatment Modality	Description
Pirfenidone	Oral medication that reduces fibroblast activity and pro-inflammatory cytokines.
Nintedanib	Oral medication that inhibits multiple tyrosine kinases involved in fibroblast proliferation.
Oxygen Therapy	It provides supplemental oxygen to improve oxygenation and relieve symptoms of hypoxemia.
Pulmonary Rehabilitation	Comprehensive programs, including exercise training, education, and support to improve lung function and overall well-being.
Lung Transplantation	It is considered for patients with advanced idiopathic pulmonary fibrosis who meet specific criteria to improve survival and quality of life.

In the next section, we will be going over these treatments. 

Current treatment & success

Antifibrotic Medications

Currently, only two drugs have been approved to be used as appropriate pharmacotherapy for IPF. 

Pirfenidone: The first trial was CAPACITY. The trials enrolled a total of 779 patients with IPF. The CAPACITY trials consisted of two Phase III randomized, double-blind, placebo-controlled trials: CAPACITY 1 and CAPACITY 2. These trials evaluated the efficacy and safety of pirfenidone in patients with IPF. Results from these trials demonstrated that pirfenidone reduced the decline in FVC compared to placebo. Additionally, pirfenidone was associated with a reduction in disease progression and improved progression-free survival compared to placebo [24]. 

The next trial conducted was ASCEND. The trial enrolled a total of 555 patients with IPF. The ASCEND trial was a Phase III randomized, double-blind, placebo-controlled trial conducted in multiple countries. This trial evaluated the efficacy and safety of pirfenidone in a broader population of patients with IPF, including those with more advanced disease. Pirfenidone-treated patients experienced a slower decline in FVC compared to placebo. Pirfenidone was associated with improved progression-free survival and slower disease progression. The ASCEND trial confirmed the findings of the CAPACITY trials and further supported the use of pirfenidone in IPF [25]. 

The RECAP trial was an open-label extension study, which further showed pirfenidone is beneficial to use in IPF and has a tolerable safety profile [26]. 

Overall, the evidence from multiple clinical trials, including the CAPACITY, ASCEND, and RECAP trials and many more, along with real-world studies, supports the use of pirfenidone as an effective treatment option for patients with IPF. 

Nintedanib: The first clinical trial supporting the use of nintedanib was the TOMORROW trial. The trial involved a total of 432 patients with IPF. It was a phase IIb randomized, double-blind, placebo-controlled trial. The trial aimed to evaluate nintedanib's impact on IPF progression, particularly on FVC. Results showed nintedanib significantly slowed FVC decline compared to placebo, indicating its beneficial effect on lung function [27]. 

The next trial is INPLUSIS. INPULSIS trials collectively enrolled 1,058 patients with IPF. The trials consisted of two phase III randomized, double-blind, placebo-controlled trials: INPULSIS-1 and INPULSIS-2. The purpose of this trial was to assess the impact of nintedanib on disease progression, particularly on the decline in FVC, in patients with IPF. The trials demonstrated that nintedanib significantly reduced the annual decline in FVC compared to placebo. Nintedanib also reduced the risk of acute exacerbations and disease progression in patients with IPF [28]. 

The study was expanded into the INPLUSIS-ON trial. This was an open-label extension study, which followed patients who completed the INPLUSIS trial. Results from this further supported the use of nintedanib [29]. 

Other key trials included INMARK and INBUILD. Together all these trials supported the use of nintedanib for IPF. [30, 31] 

Both pirfenidone and nintedanib are excellent medications currently used in patients with IPF, as evidenced by multiple trials supporting both. There is, however, a lack of studies comparing both drugs; due to which neither of them can be said to be superior to the other. 

*Oxygen* 

There is a lack of high-quality studies evaluating the efficacy of oxygen in patients with IPF and for ILD in general. Most patients with IPF will develop hypoxemia as the disease progresses initially with exertion and then with rest. The use of oxygen therapy is appropriate for these patients. There is evidence that supplemental oxygen improves exercise capacity and quality of life in patients with ILD [32]. 

*Pulmonary Rehabilitation* 

Pulmonary rehabilitation is a comprehensive program of exercise training, education, and behavioral interventions designed to improve the physical and psychological well-being of individuals with chronic respiratory diseases, including but not limited to chronic obstructive pulmonary disease (COPD), IPF, and bronchiectasis. Broadly the components of pulmonary rehabilitation include exercise training, education about the disease, behavioral interventions to achieve a better lifestyle, and psychological support. A Cochrane review summarizing multiple trials showed that pulmonary rehabilitation in patients with IPF led to an improvement in six-minute walk test results, higher peak work capacity, lower dyspnea, better quality of life, and improved survival [33].

*Lung Transplant* 

Generally, the mainstay has been to perform single lung transplants, studies have shown that bilateral lung transplants may lead to significantly higher survival times in younger patients, and better symptomatic improvement in everyone compared to single lung recipients [34, 35]. 

Table 2 summarizes the criteria for transplant referral for placement on the list [36]

**Table 2:** Criteria for transplant referral and placement on the list

Referral for Transplant:
Histologic or radiographic evidence of usual interstitial pneumonia (UIP)
Diffusing capacity for carbon monoxide (DLCO) <40% of predicted or FVC <80% of predicted.
Dyspnea or functional limitation attributed to lung disease.
Oxygen desaturation below 89% saturation, particularly during exertion.
Placement on the Transplant List:
Forced vital capacity (FVC) decline ≥10% or DLCO decline ≥15% over six months.
Oxygen desaturation to <88% or decline in distance walked in the six-minute walk test
Pulmonary hypertension confirmed by right heart catheterization or echocardiogram.
Hospitalization due to respiratory decline, pneumothorax, or acute exacerbation.

However, lung transplantation comes with its own drawbacks, including rejection, lifelong immunosuppressive therapy, and frequent follow with a healthcare professional. Despite all the efforts to make lung transplants possible, there will be many potential candidates who may never end up getting transplants due to a long waiting list.

New treatment & success

There are various treatments that are currently being studied to limit the progression of IPF including PDE4B inhibitors, combination treatment, and Pamrevlumab. 

*PDE4B Inhibitors* 

A recent study conducted in 2022 was published determining the success of PDE4B inhibition in mitigating the decline of lung function in patients diagnosed with IPF. This study that took place over the span of 12 weeks showed positive outcomes regarding the use of PDE4B inhibitors, either as standalone therapy or as part of a comprehensive combination regimen. patients who had not previously received antifibrotic treatment were administered PDE4B inhibitors. They demonstrated a substantial positive change in FVC, with an increase of 5.7 mL compared to a stark decline of -81.7 mL observed in the placebo group. Similarly, in patients concurrently taking antifibrotic medications such as nintedanib or pirfenidone, an improvement of 2.7 mL in FVC was observed in contrast to the placebo group's decline of -59.2 mL. The most common adverse effect was diarrhea. The study establishes the efficacy of PDE4B inhibitors in curbing disease progression over the 12-week period, whether administered alone or in conjunction with antifibrotic therapy. This highlights the potential of PDE4B inhibition as a promising avenue for the management of IPF, offering hope for improved patient outcomes [37]. 

*Pirfenidone and Nintedanib Combination* 

A comprehensive study conducted in Seoul investigated the efficacy of a novel treatment approach for IPF over a six-month period. This approach involved the utilization of a combination therapy comprising Pirfenidone and Nintedanib, with Nintedanib being employed as an adjunct to Pirfenidone therapy. The study demonstrated a significant decrease in the rate of decline of FVC among patients. The initial rate of FVC decline, which stood at -17.7 mL/month, exhibited a marked improvement, reducing to -10.6 mL/month after six months of treatment. The DLCO, on the other hand, did not demonstrate a significant change. It is imperative to acknowledge that a notable proportion of patients participating in this study discontinued one of the antifibrotic agents due to the occurrence of adverse reactions, manifesting predominately as anorexia and diarrhea. Moreover, comparative analysis with monotherapy did not reveal a significant disparity in outcomes. This study underscores the promising prospects of combination therapy as a potential future avenue for the treatment of IPF, albeit necessitating further investigation to elucidate its optimal utilization and long-term efficacy [38]. 

*Pamrevlumab* 

The PRAISE trial conducted an in-depth investigation into the efficacy of pamrevlumab as a therapeutic intervention for IPF. Pamrevlumab, an antibody targeting connective tissue growth factor (CTGF), acts by preventing its interaction with cytokines, thus impeding fibrosis and tissue remodeling processes. Following a 48-week treatment regimen (approximately 11 months), there was a significant decline in disease progression. This is evidenced by substantially lower quantitative lung fibrosis scores on HRCT scans compared to those receiving a placebo, with a remarkable 36.7% absolute difference noted. Importantly, the therapeutic efficacy of pamrevlumab was found to be comparable to established treatments such as nintedanib or pirfenidone yet is distinguished by a notably reduced incidence of adverse events. This promising outcome positions pamrevlumab as a prospective treatment option for individuals experiencing adverse effects from other IPF medications, offering renewed hope for improved management of this debilitating condition [39]. 

Possible future treatments 

*Transforming Growth Factor-Beta 1* 

TGF-β1 plays a pivotal role in the intricate pathogenesis of IPF. Its function revolves around the stimulation of tissue transformation, thereby facilitating fibrogenesis within the pulmonary microenvironment. This process is predominantly orchestrated through the activation of the Smad Pathway, which subsequently promotes myofibroblast differentiation, and transition of AECs to mesenchymal cells, and culminates in the intricate cascade of pulmonary fibrogenesis [40]. 

Nimbolide

Nimbolide is undergoing investigation as an epithelial-to-mesenchymal transition (EMT) inhibitor aimed at mitigating pulmonary fibrosis by disrupting the associated pathway cascade. It effectively inhibits TGF-β1-induced cell proliferation and migration, while also limiting the progressive accumulation of collagen. Overall, these results suggest that Nimbolide holds promise as an antifibrotic therapeutic intervention for attenuating PF by addressing various pathological mechanisms driving fibrosis progression [41]. 

*Stem Cells* 

Stem cells are cells that can renew and have the potential to differentiate into multiple cell lines. Stem cells are already widely used via transplantation to manage conditions like leukemia. While trials are currently only evaluating the safety of stem cell use or are only being used in trials with very few patients. 

The AETHER trial is a phase I study that investigates the use of mesenchymal stem cells in nine patients with IPF, followed over a period of 60 weeks. By 60 weeks post-infusion, there was a 3.0% mean decline in the percentage of predicted FVC and a 5.4% mean decline in the percentage of predicted DLCO [42]. 

Another study investigates the safety profile of even higher dose stem cell infusion, the results of which showed a promising safety profile [43]. 

With multiple early phases, trials are already underway. The use of stem cells as a potential treatment consideration for IPF is important to note. 

Discussion

IPF raises challenges in clinical management due to its progressive nature and limited treatment options. The current therapeutic options for IPF primarily revolve around antifibrotic medications, including pirfenidone and nintedanib, which have demonstrated efficacy in slowing disease progression and preserving lung function. These medications target key pathways involved in fibrosis and inflammation, offering patients a chance to delay disease progression and improve quality of life. 

Pirfenidone and nintedanib have changed the management of IPF by providing healthcare providers with medicines to address the underlying pathophysiology of the disease. However, despite their beneficial effects, these medications are not curative and may only slow disease progression rather than stop it from progressing altogether. Additionally, they are associated with potential side effects and may not be suitable for all patients, highlighting the need for alternative treatment options. 

Looking ahead, ongoing research and clinical trials hold promise for the development of novel therapies that could further improve the management of IPF. Emerging treatment modalities include stem cell therapy, immunomodulatory agents, and novel pharmacological targets such as PDE4B inhibitors. Stem cell therapy offers the potential to repair damaged lung tissue and modulate the immune response, while immunomodulatory agents aim to reduce inflammation and fibrosis in the lungs. 

## Conclusions

In conclusion, while the current treatment options for IPF have significantly advanced our ability to manage the disease, there remains an unmet need for effective therapies that can halt or reverse fibrotic lung damage. Ongoing research efforts and clinical trials offer hope for the development of innovative treatments that could transform the management of IPF and improve outcomes for patients in the future. Collaboration between clinicians, researchers, and pharmaceutical companies will be essential in driving progress and addressing the evolving needs of patients with IPF. 
